# KRAS mutations as architects of the tumor immune microenvironment: implications for combination therapies

**DOI:** 10.3389/fonc.2026.1933939

**Published:** 2026-08-24

**Authors:** Vasudevan Ramachandran, Haily Liduin Koyou, Siddarth Raajasekar, Mohd Hazmi Bin Mohamed, Liyana Najwa Binti Inche Mat, Venkataramanan Swaminathan, Mohd Nazil Salleh, Muhammad Evy Prastiyanto, Nurul Aini Binti Ahmad

**Affiliations:** 1 Department of Medical Sciences, Faculty of Health Sciences, MAIWP International University, Taman Batu Muda, Batu Caves, Kuala Lumpur, Malaysia; 2 Department of Conservative Dentistry and Endodontics, Saveetha Dental College and Hospitals, Saveetha Institute of Medical and Technical Sciences, Saveetha University, Chennai, India; 3 Department of Biotechnology, KIT-Kalaignar Karunanidhi Institute of Technology, Coimbatore, Tamil Nadu, India; 4 Department of Otorhinolaryngology, Head and Neck Surgery, Faculty of Medicine and Health Sciences, Universiti Putra Malaysia, Serdang, Selangor, Malaysia; 5 Department of Neurology, Faculty of Medicine and Health Sciences, Universiti Putra Malaysia, Serdang, Selangor, Malaysia; 6 Department of Diagnostic & Allied Health Sciences, Management & Science University, University Drive, Off Persiaran Olahraga, Shah Alam, Selangor, Malaysia; 7 Chancellery Office, Saito University College, Jalan, Taman Pertama, Kuala Lumpur, Malaysia; 8 Department of Medical Laboratory Technology, Faculty of Nursing and Health Sciences, Universitas Muhammadiyah Semarang, Semarang, Central Java, Indonesia

**Keywords:** immune evasion, immunotherapy, KRAS, PD-L1, tumor microenvironment

## Abstract

Oncogenic *KRAS* mutations rank among the most prevalent driver alterations in human malignancies, reaching near-universal frequency (~98%) in pancreatic ductal adenocarcinoma (PDAC) and high prevalence in colorectal cancer (CRC, ~52%) and lung adenocarcinoma (LAC, ~32%). Beyond their canonical roles in promoting cell-intrinsic proliferation and survival through the MAPK/ERK and PI3K/AKT cascades, KRAS mutations actively sculpt a profoundly immunosuppressive tumor microenvironment (TME), which constitutes a major barrier to both targeted therapy and immunotherapy. Through coordinated programs encompassing inflammatory cytokine secretion, downregulation of antigen presentation machinery, tumor-associated macrophage (TAM) reprogramming, myeloid-derived suppressor cell (MDSC) expansion, and PD-L1 upregulation, KRAS-mutant tumors establish robust immune exclusion. These programs are further stratified by co-mutations in *STK11*, *KEAP1*, and *TP53*, which define distinct immune phenotypes ranging from inflamed to profoundly immune-excluded “cold” tumors. The recent approval of covalent KRAS G12C inhibitors, sotorasib and adagrasib, has revealed that targeted KRAS blockade can remodel the TME toward an immunostimulatory state, providing a mechanistic rationale for combining KRAS-directed agents with immune checkpoint blockade, STING agonists, and neoantigen vaccines. This mini-review synthesizes the current knowledge of KRAS-immune crosstalk, highlights existing controversies and research gaps, and evaluates emerging combination strategies designed to convert immune exclusion into durable anti-tumor immunity.

## Introduction

The *KRAS* proto-oncogene encodes a small guanosine triphosphate (GTPase) that functions as a binary molecular switch, cycling between inactive guanosine diphosphate-bound and active guanosine triphosphate (GTP)-bound conformations in response to upstream signals from receptor tyrosine kinases (RTKs) and guanine nucleotide exchange factors (GEFs). Point mutations at codons 12, 13, or 61 impair intrinsic GTPase activity, locking KRAS in a constitutively active state that perpetually drives MAPK/ERK and PI3K/AKT signaling pathways. The KRAS isoform accounts for approximately 86% of all RAS mutations in cancer, with PDAC (~98%), CRC (~52%), and LAC (~32%) being the most clinically affected tumor types (1–3). For nearly four decades, KRAS has been considered “undruggable” owing to its picomolar GTP affinity and the absence of drug-binding pockets amenable to pharmacological intervention. This view was upended by the discovery of a cryptic allosteric pocket in the Switch II region of KRAS G12C, which led to the FDA approval of sotorasib (2021) and adagrasib (2022), the first direct KRAS inhibitors to enter clinical practice (4–6). However, monotherapy objective response rates of ~37–43% and median PFS of approximately 6.5 months underscored that cell-intrinsic KRAS blockade alone is insufficient for durable disease control (4–6). A growing body of evidence reveals that oncogenic KRAS does not simply accelerate tumor cell proliferation; it actively engineers an immunologically hostile TME characterized by immune exclusion, myeloid-driven suppression, and checkpoint exploitation. This realization has shifted the therapeutic paradigm toward combination strategies that simultaneously disable KRAS-driven oncogenesis and reverse KRAS-dependent immunosuppression (1, 7). This mini-review provides a focused overview of KRAS-immune crosstalk, discusses controversies and current research gaps, and evaluates the most advanced and promising combination approaches for KRAS-mutated cancers.

## KRAS-driven immunosuppressive programs

### Cytokine and chemokine remodeling

Oncogenic KRAS constitutively activates NF-κB and MAPK-dependent transcriptional programs that drive the production of pro-tumorigenic cytokines and chemokines, including IL-1α/β, IL-6, CXCL1, CXCL5, CCL2, and GM-CSF, which collectively recruit and polarize immunosuppressive myeloid populations (8, 9). In KRAS G12D-driven lung adenocarcinoma, IL-1β is markedly elevated in tumors and patient sera, and its genetic or pharmacological blockade significantly reduces tumor burden and promotes an anti-tumor immune phenotype in preclinical models (10). KRAS-mutant PDAC cells also shed exosomal packages that are taken up by macrophages via AGER-dependent mechanisms, reprogramming them through STAT3-mediated fatty acid oxidation toward an immunosuppressive M2 phenotype (11).

### Suppression of antigen presentation and innate immune sensing

A cardinal mechanism of KRAS-driven immune evasion is the suppression of MHC class I surface expression and the antigen presentation machinery (APM) (12). Common KRAS mutations suppress MHC-I levels through ERK-dependent impairment of antigen processing and IFN signaling, with restoration of MHC-I observed upon RAS inhibition in human colorectal cancer cell lines. KRAS also suppresses type I interferon (IFN) pathway activity, a program maintained across multiple co-mutation contexts, including *TP53* and *STK11*, and restoration of type I IFN signaling converts cold tumors into immunologically hot tumors responsive to ICB (13, 14). Notably, this IFN pathway suppression is established early in tumorigenesis and sustained throughout progression (14).

### PD-L1 upregulation and checkpoint exploitation

Oncogenic KRAS transcriptionally upregulates PD-L1 (CD274) expression through multiple downstream mechanisms, including ROS-mediated FGFR1 signaling and MAPK pathway activity (15, 16). MAPK pathway activity is a key determinant of PD-L1 expression in KRAS-mutant lung cancer, with co-occurring mutations in *STK11* and *KEAP1* modulating PD-L1 levels in opposite directions (15). KRAS signaling also negatively regulates IDO1 expression while enhancing PD-L1, suggesting that KRAS-mutant tumors may preferentially rely on the PD-1/PD-L1 axis rather than the IDO pathway for immune escape (15, 17, 18).

### MDSC expansion, Treg induction, and CD8+ T cell exhaustion

**Figure 1:**
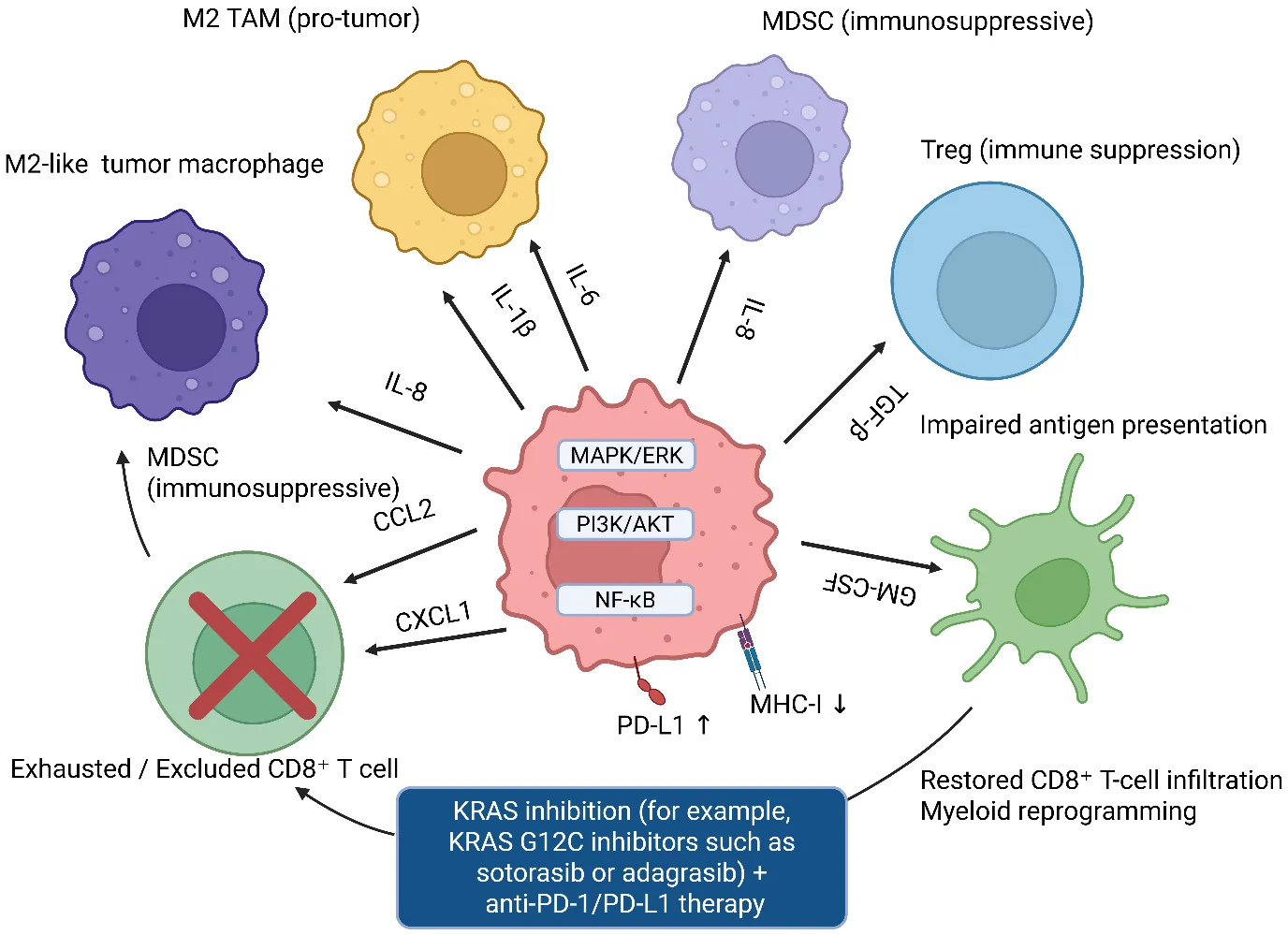
KRAS-mutant cancer cells orchestrate an immunosuppressive tumor microenvironment. A central KRAS-mutant tumor cell signals through MAPK/ERK, PI3K/AKT, and NF-κB pathways, leading to the secretion of inflammatory cytokines and chemokines (IL-1β, IL-6, IL-8, TGF-β, GM-CSF, CCL2, and CXCL1). These soluble factors recruit and polarize tumor-associated macrophages toward an M2-like phenotype, expand myeloic-derived suppressor cells (MDSCs) and regulatory T cells (Tregs), and promote the exclusion and functional exhaustion of CD8^+^ cytotoxic T cells at the tumor margin. In parallel, oncogenic KRAS downregulates MHC class I antigen presentation and upregulates PD-L1 on tumor cells, further impairing effective T-cell–mediated immunity. Pharmacologic KRAS inhibition (for example, KRAS G12C inhibitors such as sotorasib or adagrasib) in combination with anti-PD-1/PD-L1 therapy can partially reverse these programs, restoring type I interferon signaling, increasing MHC-I expression, enhancing CD8^+^ T-cell infiltration, and shifting the myeloid compartment toward a more pro-inflammatory, anti-tumor state.

MDSCs represent a major immunosuppressive population that is expanded by KRAS-driven cytokine programs, including CCL2 and CXCL1. KRAS-driven intratumoral heterogeneity promotes the infiltration of M2-polarized CD163+ macrophages through the circHIPK3/PTK2 immunosuppressive circuit, with CD163+ density correlating with mutant KRAS status and worse recurrence-free survival (19). In colorectal cancer, KRAS-driven lactic acid accumulation sensitizes cytotoxic T cells to activation-induced cell death, depleting functional CD8+ T cell responses, even when T cells successfully penetrate the tumor parenchyma. Tregs (CD4+FoxP3+), regulatory B cells (CD19+IL-10+), and Th17 cells are all expanded in KRAS-mutant tumors, contributing to multi-layered immune suppression (3, 20, 21). **Figure 1** illustrates the KRAS-driven immunosuppressive programs and principal combination strategy categories.

## The role of co-mutations in shaping KRAS-driven immune phenotypes

The immune phenotype of KRAS-mutant tumors is not uniform but is strongly shaped by co-occurring mutations that stratify patients into distinct immunological categories with differing prognoses and treatment responsiveness. The three most clinically significant co-mutations *STK11* (LKB1), *KEAP1*, and *TP53* modify KRAS-driven immune programs in mechanistically distinct and therapeutically important ways (3, 22, 23). Loss of *STK11*/LKB1 promotes a profoundly “cold” tumor phenotype through CXCL7-mediated neutrophil recruitment and suppression of STING innate immune signaling, rendering tumors refractory to PD-1/PD-L1 inhibitors and establishing poor prognosis, even in the context of KRAS G12C inhibitor therapy (24). Co-occurring *KEAP1* mutations further exacerbate immune evasion through enhanced oxidative stress resistance and additional impairment of the antigen processing machinery, with the KRAS + *STK11* + *KEAP1* triple co-mutation status representing the most immune-excluded and therapeutically challenging subgroup (17, 23). In contrast, KRAS-mutant tumors co-mutated with *TP53* retain a more inflammatory, T cell-infiltrated phenotype with a higher tumor mutational burden (TMB), correlating with comparatively better, though still incomplete, responses to immune checkpoint inhibitors. At the allele level, KRAS G12R-mutant PDAC tumors display higher infiltration of CD8+ T cells, CD4+ T cells, and B cells compared to G12D, suggesting that allele-specific immune profiling may eventually inform immunotherapy selection beyond current G12C versus non-G12C stratification (3, 17, 22, 24). **Table 1** summarizes the immune landscape and ICI responsiveness associated with key co-mutations and allelic contexts.

**Table 1:** Co-mutations stratify KRAS-mutant tumor immune phenotypes and ICI response.

Context	Immune phenotype	TME characteristics	ICI response	Reference
KRAS + *TP53*	Inflamed/”hot”	Higher TIL infiltration, elevated TMB, PD-L1+	Improved	(3)
KRAS + *STK11*	Cold/immune desert	T cell exclusion, neutrophil infiltration, low PD-L1, low TMB	Resistant	(22)
KRAS + *KEAP1*	Cold/suppressed	Oxidative resistance, impaired APM	Resistant	(17, 22)
KRAS + *STK11* + *KEAP1*	Profoundly cold	Maximal immune exclusion, worst prognosis	Highly resistant	(17)
KRAS G12D (PDAC)	Immunosuppressive	Low PD-L1, MDSC-rich, T cell-poor	Very poor	(24, 25)
KRAS G12R (PDAC)	Relatively inflamed	Higher B cell, CD8+, CD4+ T cell infiltration	Potentially better	(24)

## KRAS inhibition remodels the tumor immune microenvironment

Sotorasib and adagrasib were developed as anti-proliferative targeted agents but rapidly demonstrated potent immunomodulatory effects beyond cell-autonomous tumor cell killing (4). In immunocompetent syngeneic mouse models, sotorasib treatment increases CD8+ T cell infiltration, enhances IFN-γ production, and fosters a pro-inflammatory TME. In combination with anti-PD-1, it produces complete regression in 9 of 10 mice compared to 1 of 10 with monotherapy alone. Adagrasib (MRTX849) reconditions the TME by increasing CD8+ T cell numbers, decreasing M2-polarized macrophages, reducing MDSCs and Tregs, and enhancing immunogenic cell death through the restoration of type I IFN signaling (20, 26). Spatially, KRAS G12C inhibition converts immune-excluded tumors with T cells accumulating at the invasive margin into immune-infiltrated tumors with deep parenchymal T cell penetration (4, 6, 20).

A mechanistically unique observation is that covalent KRAS G12C inhibition induces the presentation of inhibitor-modified hyphenated peptide neoepitopes on MHC class I complexes, effectively generating drug-modified neoantigens that tag residual tumor cells for immune destruction, creating a *de novo* vaccination-like event concurrent with targeted therapy (27, 28). The KRAS G12D-selective inhibitor MRTX1133 similarly induces a proinflammatory TME enriched in M1 macrophages and cytotoxic T cells in PDAC models, and a fully allele-agnostic mutant-KRAS inhibitor has been demonstrated to suppress tumor immunosuppression in early preclinical studies (27, 28).

## Discussion

### Controversies and competing perspectives

Several key controversies currently shape the KRAS-immunity field. The first concerns whether KRAS inhibitor + ICB combinations deliver additive benefit beyond what ICB or KRAS inhibition achieves alone in unselected patients (5, 26, 29). Preclinical synergy has been consistently demonstrated across syngeneic models; however, early clinical combination signals have been complicated by overlapping hepatotoxicity and a lack of clear PD-L1 or TMB predictive enrichment (5, 6). A counterargument holds that KRAS G12C inhibitors should be evaluated sequentially with ICB rather than concurrently, based on pharmacodynamic modeling suggesting that KRAS-inhibitor-driven TME remodeling may take 4–8 weeks to fully manifest implying that upfront combination may not be the optimal dosing strategy (6, 26, 29).

A balanced assessment of the controversy requires a distinction between three claims which are often conflated: concomitant administration is biologically synergistic, clinically acceptable, and observed toxicity is a class effect. The preclinical case for concurrency is internally consistent but of limited applicability. Syngenic models have shown reproducibly that inhibition of KRAS G12C increases CD8+ T cell infiltration and predisposes tumors to PD-1 blockade, but these studies were conducted in murine transplantable, immunologically susceptible tumors with a high mutational burden, which lack the immune exclusion and co-mutational complexity of human KRAS-mutant disease (30, 31). Clinical experience has been correspondingly inconsistent. In the CodeBek 100 and 101 dose escalation cohorts, sotorasib in combination with pembrolizumab or atezolizumab resulted in Grade 3–4 treatment related adverse events, mostly transaminase elevations, in 58 patients, with a confirmed response rate of 29%, which is below the activity of sotorasib alone, although the responses were shown to be durable (median duration of 17.9 months). Critically, the presence of sotorasib for 21–42 days prior to initiation of checkpoint inhibitor therapy reduced the incidence of Grade 3–4 events (53% versus 74% in the pembrolizumab cohorts) and treatment discontinuation (32% versus 53%). This observation is the main empirical support for staged rather than concurrent treatment (30).

But two further observations fundamentally complicate any easy conclusion that sequential management is a safer strategy. First, postponing immunotherapy does not cancel out this interaction, but may in fact reverse it. In a retrospective study of 102 patients who received sotorasib outside clinical trials, the proportion of patients who received anti-PD-(L)1 therapy as the immediate prior treatment was 50% (p<0.001) and the proportion of patients who experienced severe hepatotoxicity was 33% (p<0.006) and the investigator recommended a 30-day interval after the last dose of treatment. As the pharmacokinetic tail of antibodies to PD-(L)1 extends over several weeks, nominally sequential administration is immunologically consistent. Secondly, toxicity is not a class effect and therefore does not provide a reliable basis for general scheduling rules. In the phase 2 portion of the KRYSTAL-7 trial, concomitant first-line administration of adagliflozin plus pembrolizumab resulted in an objective response rate of 44% and a disease control rate of 81% (32). Elevations of aminotransferase were frequent (39.6 and 35.1%, respectively, grade 3-4), but discontinued in 0.4 and 14.7% of patients (32). The difference between sotorasib and adagrasib in the same disease and in the same partner antibody is more likely to be attributed to the different pharmacokinetic profiles, the longer half-life and the lower peak-to-trough ratio of adagrasib compared to sotorasib, and to the different off-target cysteine reactivity than to any general property of the blockade of the KRAS pathway. The operative question is therefore not whether the combinations should be simultaneous or sequential in the abstract, but which agent, when and how the exposure is to be determined, and which interval between them. It is not possible to make an assessment of the current evidence where no randomized comparison of treatment regimens has been made and where efficacy conclusions are based on cross-trial comparisons of non-randomized cohorts that differ in treatment line, PD-L1 distribution and prior immunotherapy exposure.

The second controversy concerns the predictive value of PD-L1 expression in KRAS-mutant tumors. Unlike KRAS-wild-type NSCLC, where PD-L1 TPS ≥50% strongly predicts pembrolizumab benefit, PD-L1 expression in KRAS G12C-mutant NSCLC does not consistently correlate with the benefit from KRAS inhibitor + ICB combinations, challenging the assumption that KRAS inhibitor-driven PD-L1 upregulation translates into improved ICI responsiveness (5, 16, 20, 29, 33). This suggests that pathway-level immune profiling encompassing co-mutation status, spatial T cell infiltration patterns, IFN-γ gene signatures, and TCR diversity may ultimately supersede PD-L1 as the dominant biomarker.

Clinical evidence supporting alternative treatment for PD-L1 is strongest in co-mutational setting. In a Stand Up to Cancer cohort study of 174 patients with KRAS-mutated lung adenocarcinoma, the Objective Response Rate (ORR) to PD-1 blockade was 7.4% for KRAS and STK11, 35.7% for KRAS and STK53, and 28.6% for KRAS alone (p<0.001) (study number: CHEM-057). Decisive for this argument, the only variable significantly associated with PD-L1 negativity in tumors with intermediate or high TMB was STK11 and LKB1 changes in 924 lung adenocarcinomas, and its negative effect on the outcome of checkpoint inhibitors was maintained in the PD-L1 positive population (34). Thus, the genotype associated with the co-mutations is not only correlated with the expression of PD-L1; it also stratifies the benefit orthogonally, which is exactly what the additional biomarker needs to do. The relevant conclusion is not that the PD-L1 score is not informative in KRAS-mutant disease, but that it is not sufficient in its own right, a conclusion supported by the KRYSTAL-7 biomarker-based analysis, which showed that the response to adagliflozin plus pembrolizumab was still increasing monotrophically across all the PD-L1 classes (36% at TPS < 1, 41% at TPS 1 to 49% and 61% at TPS > 50%) (32).

The transcriptional and spatial data address different constraints, in that immunohistochemistry of PD-L1 tests a single protein in a single cell compartment at a single location in the universe. A profile of T cell-inactivated gene expression, anchored to IFN-γ-responsive genes that control antigen presentation, chemokine expression and cytotoxicity, has been derived and validated in nine indications and has been confirmed with a discriminatory effect of pembrolizumab against PD-L1 immunohistochemistry independently in head and neck squamous cell carcinoma (35). As oncogenic KRAS suppresses interferon type I and type II signaling as the primary evasion program, the IFN-γ signature is mechanistically more appropriate for the biology under investigation than PD-L1, which is expressed in this setting as a partial cellular intranscription and not as a marker of pre-existing adaptive immunity (15, 16, 36). Spatial profiling offers dimensions missing in the previous two methods. A systematic review and meta-analysis of 8,135 patients with over 10 different solid tumor types found that multiplex immunohistochemistry and immunofluorescence were more predictive of anti-PD-1 and anti-PD-L1 responses than individual immunohistochemistry and TMB or gene expression profiling, and multi-drug combinations were more predictive (37). This is particularly relevant for KRAS-mutant tumors where the defining lesion is more structural than quantitative, T cells accumulate at the invasive margin rather than being absent, and inhibition of KRAS G12C acts by redistribution rather than simply by expansion of the intratumoral compartment of the T cells (38). However, none of these assays were prospectively qualified as predictive biomarkers in randomized, controlled, combination studies with KRAS. Each of these methods is derived from a retrospective or multidimensional dataset of tumors. In particular, multiplex imaging remains limited in terms of test standardization, tissue adequacy and interlaboratory reproducibility. Gene expression signatures from single site biopsies of spatially heterogeneous tumors are subject to sampling bias. Therefore, a composite classifier integrating co-mutational genotypes, the transcription score of the interferon pathway and spatial T cell topology is a realistic short-term goal, rather than substituting one biomarker for another.

The third point of debate involves allele-specific versus allele-agnostic therapeutic strategies. While the G12C-centric drug development trajectory has yielded FDA-approved agents, KRAS G12D is the dominant allele in PDAC, the most lethal and immunologically cold KRAS-mutant cancer, and emerging G12D inhibitors, including MRTX1133 and RMC-9805, are entering clinical development with immunological profiling as an embedded endpoint (27, 39).

## Current research gaps

Despite these rapid advances, several important gaps remain unresolved.

- Non-G12C allele TME profiling: Systematic, allele-resolved immune landscape characterization (G12D, G12V, G12R, G13D) across tumor types is lacking, with most mechanistic TME studies conducted in G12C NSCLC models. Allele-specific immunogenicity differences may be clinically meaningful, particularly for vaccine and adoptive T cell therapy designs (24, 39).
- Temporal dynamics of TME remodeling: The kinetics by which KRAS inhibition remodels the TME and the optimal timing window for introducing immunotherapy have not been prospectively characterized in clinical studies. Single time-point biopsy analyses cannot capture the dynamic immune reprogramming that occurs over weeks, as suggested by preclinical models (6, 20).
- Co-mutation-targeted strategies: No approved or late-stage strategy exists specifically for the KRAS + *STK11* or KRAS + *KEAP1* patient subset, which constitutes approximately 20–30% of KRAS-mutant NSCLC and represents the most immunotherapy-refractory group. STING agonist-based approaches offer theoretical promise but lack dedicated clinical validation in this context (13, 22).
- Biomarker validation beyond PD-L1: Prospective co-mutation profiling, spatial immune phenotyping, and IFN-γ gene expression signatures require validation as predictive biomarkers in dedicated KRAS-stratified combination trials (23, 33).

## Implications of non-G12C allele gaps for therapeutic development

- The consequences of these gaps go beyond descriptive incompleteness, since the G12C-centric evidence base is used, to a large extent by default, as a template for the evolution of alleles that differ in both biochemistry and immunobiology. Three specific translational risks are identified: first, the mechanism by which covalent G12C inhibitors induce immunogenicity is allele-dependent: haptenation of mutant cysteine results in the formation of peptides that are modified by the inhibitor, which are of class I of the *de novo* neoantigen, G12D, G12V or G13D, and which are not susceptible to the same covalent targeting (28). Therefore, it cannot be assumed that combinations based on drug induced generation of neoepitopes are transmitted between alleles. Second, the co-mutational structure that controls the immune phenotype is specific to the tumor type and the STK11 and KEAP1 framework in lung adenocarcinoma, where G12D is predominant and where the major immunosuppressive determinants are stromal and myeloid factors (22, 24, 25), is not well established (22). Therefore, the transfer of patient selection in patients with concurrent mutations from NSCLC to pancreatic disease is currently not supported. Thirdly, the available allele-related immunological data are observational and derived from bulk profiling of untreated subjects; for example, the relatively inflammatory phenotype reported for G12R in PDAC versus G12D indicates a baseline difference but not a difference in therapeutic conversion potential, which is a property that should inform the design of the study (24).
- The issue of allele-specific versus allele-agnostic treatment has recently gained empirical weight, changing the terms of the debate. In a randomized, controlled Phase III study, RASolute 302, the oral, multi-selective RAS inhibitor daraxonrasib, which targets the active GTP binding state of mutant and wild-type RAS, was compared with chemotherapy in 500 patients with previously treated metastatic PDAC, 91.8% of whom had RAS G12 mutations. In the overall population, the median overall survival was 13.2 versus 6.6 months and the treatment-related discontinuation rate was 61.8% versus 69.2% (hazard ratio 0.40; p<0.001) and the median progression-free survival was 7.3 versus 3.5 months (40). This is the first demonstration that allele-specific RAS-directed agents can confer survival benefits in the most immunologically hostile KRAS mutant malignancies and shows that the range of allele coverage does not have to be purchased at the price of tolerability. But it does not solve the strategic question. Allegral-agnostic inhibition necessarily involves wild type RAS in normal tissue, which imposes a target-specific therapeutic index constraint that mutant selective agents avoid and which may be the basis for the characteristic dermatological and mucosal toxicity of this class. Allelele-selective agents have the benefit of a broader therapeutic window and drug-induced neoantigen presentation in G12C. Moreover, the immunological consequences of inhibition of pan-RAS are essentially unknown. Whether suppression of wild-type RAS signaling in T cells, dendritic cells and myeloid populations enhances or hinders anti-tumor immunity is an open and controversial issue, as RAS and MAPK signaling are integral to the activation of T-cell receptors and myeloid differentiation. Addressing this issue will require immunological monitoring endpoints to be prospectively included in allele-agnostic studies, rather than inferred retrospectively. Both approaches are likely to be complementary: allele selective inhibition, where the target allele is in the permissive immune context, and alleleagnostic inhibition, where the allele diversity, subclonal variation, or acquired resistance is mediated by secondary RAS changes (39, 41).

## Emerging combination strategies and future directions

The combination of KRAS G12C inhibitors with anti-PD-1 therapy is the most clinically advanced approach, exemplified by the ongoing Phase III KRYSTAL-7 trial evaluating adagrasib plus pembrolizumab versus pembrolizumab monotherapy in treatment-naïve KRAS G12C-mutant NSCLC (PD-L1 TPS ≥50%). A triple combination of adagrasib + cetuximab + cemiplimab is under Phase 1b/2 evaluation for KRAS G12C-mutant metastatic CRC, reflecting the need for multi-target approaches in colorectal disease. Combining RAS (ON) G12C-selective inhibitors with SHP2 inhibitors, which block upstream RTK-to-RAS signal propagation and prevent adaptive resistance, further sensitizes tumors to ICB by preventing re-engagement of the immunosuppressive cytokine program after KRAS inhibition (42, 43).

Beyond checkpoint blockade, STING agonist delivery, particularly through lipid nanoparticle formulations, co-delivering mutant KRAS mRNA alongside STING activators, has demonstrated the ability to reprogram tolerogenic immune niches in PDAC liver metastasis models, converting cold to hot tumor microenvironments. Neoantigen-based immunotherapy exploiting the shared, clonal, and immunogenic nature of KRAS mutations is advancing through chemically modified mutant peptide vaccines, hapten-based immunotherapeutics, immunotherapeutics leveraging KRAS G12C inhibitor-modified MHC-I neoepitopes, and T-cell receptor (TCR)-engineered adoptive cell therapies, which achieved tumor regression in KRAS G12D-mutant PDAC in an early clinical report. Combined KRAS inhibition and adoptive immunotherapy have generated durable responses in PDAC preclinical models, supporting formal clinical investigations. Looking further ahead, the development of allele-agnostic mutant KRAS inhibitors capable of targeting G12D, G12V, and G13D across tumor types in combination with immune activation strategies tailored to the co-mutation-defined immune phenotype represents the most impactful frontier for converting KRAS-driven immune exclusion into sustained clinical benefit (25, 27, 28, 44).

## Limitations, challenges, and safety considerations

Each of the strategies outlined above has specific, and in some cases proven, liabilities that temper expectations in the short term. In checkpoint combinations, the overlapping hepatotoxicity discussed above remains the predominant limitation and is further compounded by the addition of other agents. Vertical pathway blockade by SHP2 inhibitors is mechanistically attractive, as it delays adaptive reactivation of the immunosuppressive cytokine program by RTK. However, it aggregates target toxicity of suppression of the RAS pathway, such as oedema, thrombocytopenia, diarrhea and hypertension, onto a backbone that is already associated with immune-mediated organ damage. No triplet containing SHP2 has been shown to have a dose intensity that is able to maintain continuous pathway suppression with acceptable tolerability (42, 45). The regimen of adagliflozin, cetuximab, and ceftriaxone currently being evaluated in colorectal cancer similarly superimposes EGF toxicities on this profile. A general rule of thumb emerges from these observations: since KRAS inhibition itself remodels the microenvironment towards inflammation, combinations that further increase immune activation may carry a mechanistically predictable rather than incidental risk of immunotoxicity.

The clinical experience with STING agonists provides a cautionary example of unsuccessful translation of preclinical activity. In a Phase I dose escalation study of MIW815 intratumoral cyclic dinucleotide (ADU-S100) in 47 patients, the maximum tolerated dose was not reached and systemic immune activation was demonstrated, but only one confirmed partial response was observed. Pharmacokinetic analysis revealed a rapid exit from the injection site with a terminal plasma half-life of approximately 24 min, suggesting that the limiting variable is intratumoral retention and not STING binding (46). The subsequent Phase Ib combination of spartalizumab with the anti-PD-1 antibody was delivered safely but produced minimal efficacy in refractory disease and the investigators concluded that the findings did not support a move to a Phase II or III evaluation (47). Three lessons are directly applicable to the KRAS. The main failure mode is delivery rather than target validity, which is the strongest available justification for the lipid nanoparticle and co-delivery formulations currently being investigated. Intratumoral administration is not feasible in the visceral and disseminated diseases characterized by advanced PDAC and NSCLC; therefore, systemic bioavailability agonists are required. Sustained systemic activation of STING, in turn, carries the risk of not only cytokine release toxicity but also paradoxical immunosuppression, since chronic exposure to type I interferons may induce MDSC proliferation and T-cell depletion. In the specific case of STK11 mutant tumors, where STING signaling is suppressed at the level of the tumor cells, it is not yet proven that pharmacological agonism can overcome the genetically induced pathway defect (22, 36).

Neoantigen-based approaches are limited by their applicability and probative value rather than their toxicity. A TCR-engineered adoptive therapy directed at KRAS G12D resulted in a 72% partial response after 6 months in one patient with metastatic PDAC, with engineered cells remaining in excess of 2% of circulating T cells; this is a mechanistically important result, but one that is limited by the HLA-C02 binding in a small minority of patients, reported in the US (48). Wider application will require a wider repertoire of TCRs, including multiple HLA and KRAS alleles, as well as alleviation of cytokine release syndrome and neurotoxicity, addressing production costs and logistical complexity. Peptide vaccination avoids HLA depletion, but is currently based on narrower evidence base than is generally accepted. In a final analysis of phase 1 AMPLIFY-201, a lymph node-targeted phase 2P study of 25 patients with minimal residual disease, the median relapse-free and overall survival was not achieved in patients with a T cell response to mutant KRAS-specific antibodies above 9.17 times the baseline (hazard ratios 0.12 and 0.23). These differences are striking, but they are derived from a single arm study of 25 patients, where a *post hoc* comparison is made between immunologically defined subgroups rather than randomized arms, as this analysis is prone to bias due to the burden of disease and the time to survival required to generate a measurable immune response. Therefore, a randomized Phase 2 trial with the seven-peptide formulation is required before efficacy can be considered to be established (49, 50). Finally, the assumption common to all neoantigen strategies that mutant KRAS peptides are reliably expressed on the surface of tumor cells is undermined by the KRAS-driven suppression of the antigen presenting machinery described above, which may require the simultaneous re-expression of MHC Class I, possibly via KRAS inhibition, to achieve the desired effect (12, 38).

## Conclusions

Oncogenic KRAS is not only a mitogenic agent that is controlled by cells but also a facilitator of immune evasion through cytokine-driven myeloid recruitment, suppression of antigenic presentation and interferon signaling, and checkpoint-dependent programs, which are modulated and partially reversed by pharmacological blockade of KRAS. Progress is hampered by four knowledge gaps. The optimal time relationship between KRAS inhibition and checkpoint blockade remains unclear, and is more likely to be confounded by the pharmacology of the individual agent than resolved by any randomized comparison of schedules. The development of predictive biomarkers has not progressed beyond retrospective and tumor-wide datasets for the prospectively qualification of KRAS-stratified composite classifiers. Despite the emergence of the first randomized survival data for this class, immunobiology of non-G12C alleles and in particular of allele-agnostic inhibition of RAS are largely unknown. There is no specific treatment strategy for the co-mutated subgroup STK11 and KEAP1, which is the most immunotherapy-resistant population. The most productive short-term research directions stem directly from these gaps: prospective randomized tumor samples to determine the kinetics and durability of KRAS inhibitor-induced immune remodeling in STK11-mutant disease and antigen-presentation failure in patients with KEAP1-mutant disease, and to address the immune monitoring endpoints directly rather than as retrospective correlators; biomarker-selective and allele-agnostic combinations for composite classifiers; Progress will depend less on the identification of additional combinations than on the determination, with sufficient rigor, of which combination, in which order, and for how long, the patient should be treated.
